# Correction: Service quality assessment and enhancement using Kano model

**DOI:** 10.1371/journal.pone.0293766

**Published:** 2023-10-26

**Authors:** Sharareh Kermanshachi, Thahomina Jahan Nipa, Halil Nadiri

There is an error in affiliation 3 for author Halil Nadiri. The correct affiliation 3 is: Department of Business Administration, Cyprus International University, Nicosia, North Cyprus, Turkey.
