# Supplementary material for: Service quality assessment and enhancement using Kano model
Source: PLoS One. 2022 Feb 25;17(2):e0264423. doi: 10.1371/journal.pone.0264423 (PMC8880948; doi:10.1371/journal.pone.0264423)
Supplement: S1 Appendix — (DOCX) [file pone.0264423.s002.docx]

**S1 Appendix**

Service Quality Assessment and Enhancement Using Kano Model

Survey Questions

***Survey Instruction***

Dear Participant,

We are writing to ask for your participation in a research survey at the Eastern Mediterranean University aiming to identify the attributes of the retail stores which have an impact on the customer satisfaction. Your participation in the survey is voluntary, and your response are completely confidential. This survey will take about **15 minutes**.

There are no perceived risks for participating in the survey, and you can quit the survey at any time you want. Your personal information will not be represented in the report or in any data available to the public. We will use your individual responses only for the purposes of this study. Thank you in advance for participating in this study. If you have any questions, you can contact Sharareh Kermanshachi at [Sharareh.kermanshachi@emu.edu.tr](mailto:Sharareh.kermanshachi@emu.edu.tr).

Thank you very much for your participation.

Please provide your signature in the box below as consent of your agreement to participate in this survey at your own will.

Part 1: Demographic questions

1. Please specify your age range.

( ) 17 - 20

( ) 21 - 24

( ) 25 - 28

( ) 29 - 32

( ) 33 and more

1. Please specify your gender.

( ) Female

( ) Male

1. Please specify your Institute and department:
2. Please specify the total monthly income of your family.

( ) 1500 TL and less

( ) 1501 TL - 2500 TL

( ) 2500 TL - 4000 TL

( ) 4001 TL - 6000 TL

( ) 6001 TL and more

1. Your Nationality:

Part II: Experiential Marketing

***Section A:***

By assuming your experience in a retail store. Please answer below statements according to following scale.

1. I like it that way
2. It must be that way
3. I am neutral
4. I can live with it that way
5. I dislike it that way

| **#** | **Statements** | **Perceptions**  2  3  4  5 | | | | |
| --- | --- | --- | --- | --- | --- | --- |
| 1 | If store has modern looking equipment and fixtures, how do you feel? | 1 | 2 | 3 | 4 | 5 |
| 2 | If store has physical facilities that are visually appealing, how do you feel? | 1 | 2 | 3 | 4 | 5 |
| 3 | If store has materials associated with its service (such as shopping bags, catalogs, or statements) that are visually appealing, how do you feel? | 1 | 2 | 3 | 4 | 5 |
| 4 | If store has clean, attractive and convenient public areas (restrooms, fitting rooms), how do you feel? | 1 | 2 | 3 | 4 | 5 |
| 5 | If store has layout that makes it easy for customers to find what they need, how do you feel? | 1 | 2 | 3 | 4 | 5 |
| 6 | If store has layout that makes it easy for customers to move around in the store, how do you feel? | 1 | 2 | 3 | 4 | 5 |
| 7 | If the store does something by a certain time, when it promises to do, how do you feel? | 1 | 2 | 3 | 4 | 5 |
| 8 | If store provides its services at the time it promises to do so, how do you feel? | 1 | 2 | 3 | 4 | 5 |
| 9 | If store performs the service right the first time, how do you feel? | 1 | 2 | 3 | 4 | 5 |
| 10 | If store has merchandise available when the customers want it, how do you feel? | 1 | 2 | 3 | 4 | 5 |
| 11 | If store has error-free sales transactions and records, how do you feel? | 1 | 2 | 3 | 4 | 5 |
| 12 | If store has employees that have the knowledge to answer customers’ questions, how do you feel? | 1 | 2 | 3 | 4 | 5 |
| 13 | If store has employees that their behavior instill confidence in customers, how do you feel? | 1 | 2 | 3 | 4 | 5 |
| 14 | If store makes customers feel safe in their transactions with this store, how do you feel? | 1 | 2 | 3 | 4 | 5 |
| 15 | If store has employees who give prompt service to customers, how do you feel? | 1 | 2 | 3 | 4 | 5 |
| 16 | If store has employees who can tell customers exactly when services will be performed, how do you feel? | 1 | 2 | 3 | 4 | 5 |
| 17 | If store has employees who are never too busy to respond to customers’ requests, how do you feel? | 1 | 2 | 3 | 4 | 5 |
| 18 | If store gives customers individual attention, how do you feel? | 1 | 2 | 3 | 4 | 5 |
| 19 | If store has employees that are consistently courteous with customers, how do you feel? | 1 | 2 | 3 | 4 | 5 |
| 20 | If store has employees that treat customers courteously on the telephone, how do you feel? | 1 | 2 | 3 | 4 | 5 |
| 21 | If store willingly handles returns and exchanges, how do you feel? | 1 | 2 | 3 | 4 | 5 |
| 22 | If store shows a sincere interest in solving customer’s problem, how do you feel? | 1 | 2 | 3 | 4 | 5 |
| 23 | If store has employees who are able to handle customer complaints directly and immediately, how do you feel? | 1 | 2 | 3 | 4 | 5 |
| 24 | If store offers high quality merchandise, how do you feel? | 1 | 2 | 3 | 4 | 5 |
| 25 | If store provides plenty of convenient parking for customers, how do you feel? | 1 | 2 | 3 | 4 | 5 |
| 26 | If store has operating hours convenient to all their customers, how do you feel? | 1 | 2 | 3 | 4 | 5 |
| 27 | If store accepts most major credit cards, how do you feel? | 1 | 2 | 3 | 4 | 5 |
| 28 | If store offers its own credit card, how do you feel? | 1 | 2 | 3 | 4 | 5 |

***Section B:***

By assuming your experience in a retail store. Please answer below statements according to following scale.

1. I like it that way
2. It must be that way
3. I am neutral
4. I can live with it that way
5. I dislike it that way

| **#** | **Statements** | **Perceptions**  2  3  4  5 | | | | |
| --- | --- | --- | --- | --- | --- | --- |
| 1 | If store does not have modern looking equipment and fixtures, how do you feel? | 1 | 2 | 3 | 4 | 5 |
| 2 | If store does not have physical facilities that are visually appealing, how do you feel? | 1 | 2 | 3 | 4 | 5 |
| 3 | If store does not have materials associated with its service (such as shopping bags, catalogs, or statements) that are visually appealing, how do you feel? | 1 | 2 | 3 | 4 | 5 |
| 4 | If store does not have clean, attractive and convenient public areas (restrooms, fitting rooms), how do you feel? | 1 | 2 | 3 | 4 | 5 |
| 5 | If store does not have layout that makes it easy for customers to find what they need, how do you feel? | 1 | 2 | 3 | 4 | 5 |
| 6 | If store does not have layout that makes it easy for customers to move around in the store, how do you feel? | 1 | 2 | 3 | 4 | 5 |
| 7 | If the store does not do something by a certain time, when it promises to do, how do you feel? | 1 | 2 | 3 | 4 | 5 |
| 8 | If store does not provide its services at the time it promises to do so, how do you feel? | 1 | 2 | 3 | 4 | 5 |
| 9 | If store does not perform the service right the first time, how do you feel? | 1 | 2 | 3 | 4 | 5 |
| 10 | If store does not have merchandise available when the customers want it, how do you feel? | 1 | 2 | 3 | 4 | 5 |
| 11 | If store does not have error-free sales transactions and records, how do you feel? | 1 | 2 | 3 | 4 | 5 |
| 12 | If store does not have employees that have the knowledge to answer customers’ questions, how do you feel? | 1 | 2 | 3 | 4 | 5 |
| 13 | If store does not have employees that their behavior instill confidence in customers, how do you feel? | 1 | 2 | 3 | 4 | 5 |
| 14 | If store does not make customers feel safe in their transactions with this store, how do you feel? | 1 | 2 | 3 | 4 | 5 |
| 15 | If store does not have employees who give prompt service to customers, how do you feel? | 1 | 2 | 3 | 4 | 5 |
| 16 | If store does not have employees who can tell customers exactly when services will be performed, how do you feel? | 1 | 2 | 3 | 4 | 5 |
| 17 | If store does not have employees who are never too busy to respond to customers’ requests, how do you feel? | 1 | 2 | 3 | 4 | 5 |
| 18 | If store does not give customers individual attention, how do you feel? | 1 | 2 | 3 | 4 | 5 |
| 19 | If store does not have employees that are consistently courteous with customers, how do you feel? | 1 | 2 | 3 | 4 | 5 |
| 20 | If store does not have employees that treat customers courteously on the telephone, how do you feel? | 1 | 2 | 3 | 4 | 5 |
| 21 | If store does not willingly handle returns and exchanges, how do you feel? | 1 | 2 | 3 | 4 | 5 |
| 22 | If store does not show a sincere interest in solving customer’s problem, how do you feel? | 1 | 2 | 3 | 4 | 5 |
| 23 | If store does not have employees who are able to handle customer complaints directly and immediately, how do you feel? | 1 | 2 | 3 | 4 | 5 |
| 24 | If store does not offer high quality merchandise, how do you feel? | 1 | 2 | 3 | 4 | 5 |
| 25 | If store does not provide plenty of convenient parking for customers, how do you feel? | 1 | 2 | 3 | 4 | 5 |
| 26 | If store does not have operating hours convenient to all their customers, how do you feel? | 1 | 2 | 3 | 4 | 5 |
| 27 | If store does not accept most major credit cards, how do you feel? | 1 | 2 | 3 | 4 | 5 |
| 28 | If store does not offer its own credit card, how do you feel? | 1 | 2 | 3 | 4 | 5 |

***Section C:***

By assuming your experience in a retail store. Please answer below statements according to following scale.

1. Not at all important
2. Not important
3. Slightly not important
4. Neutral
5. Slightly important
6. Important
7. Extremely important

| **#** | **Statements** | **Importance** | | | | | | |
| --- | --- | --- | --- | --- | --- | --- | --- | --- |
|  |  | Not at all important | | |  | Extremely important | | |
|  |  | 1 | 2 | 3 | 4 | 5 | 6 | 7 |
| 1 | How important it is for you that the store has modern looking equipment and fixtures. | 1 | 2 | 3 | 4 | 5 | 6 | 7 |
| 2 | How important it is for you that the store has physical facilities that are visually appealing. | 1 | 2 | 3 | 4 | 5 | 6 | 7 |
| 3 | How important it is for you that the store has materials associated with its service (such as shopping bags, catalogs, or statements) that are visually appealing. | 1 | 2 | 3 | 4 | 5 | 6 | 7 |
| 4 | How important it is for you that the store has clean, attractive and convenient public areas (restrooms, fitting rooms). | 1 | 2 | 3 | 4 | 5 | 6 | 7 |
| 5 | How important it is for you that the store has layout that makes it easy for customers to find what they need. | 1 | 2 | 3 | 4 | 5 | 6 | 7 |
| 6 | How important it is for you that the store has layout that makes it easy for customers to move around in the store. | 1 | 2 | 3 | 4 | 5 | 6 | 7 |
| 7 | How important it is for you that the store does something by a certain time, when it promises to do. | 1 | 2 | 3 | 4 | 5 | 6 | 7 |
| 8 | How important it is for you that the store provides its services at the time it promises to do so. | 1 | 2 | 3 | 4 | 5 | 6 | 7 |
| 9 | How important it is for you that the store performs the service right the first time. | 1 | 2 | 3 | 4 | 5 | 6 | 7 |
| 10 | How important it is for you that the store has merchandise available when the customers want it. | 1 | 2 | 3 | 4 | 5 | 6 | 7 |
| 11 | How important it is for you that the store has error-free sales transactions and records. | 1 | 2 | 3 | 4 | 5 | 6 | 7 |
| 12 | How important it is for you that the store has employees that have the knowledge to answer customers’ questions. | 1 | 2 | 3 | 4 | 5 | 6 | 7 |
| 13 | How important it is for you that the store has employees that their behavior instill confidence in customers. | 1 | 2 | 3 | 4 | 5 | 6 | 7 |
| 14 | How important it is for you that the store makes customers feel safe in their transactions with this store. | 1 | 2 | 3 | 4 | 5 | 6 | 7 |
| 15 | How important it is for you that the store has employees who give prompt service to customers. | 1 | 2 | 3 | 4 | 5 | 6 | 7 |
| 16 | How important it is for you that the store has employees who can tell customers exactly when services will be performed. | 1 | 2 | 3 | 4 | 5 | 6 | 7 |
| 17 | How important it is for you that the store has employees who are never too busy to respond to customers’ requests. | 1 | 2 | 3 | 4 | 5 | 6 | 7 |
| 18 | How important it is for you that the store gives customers individual attention. | 1 | 2 | 3 | 4 | 5 | 6 | 7 |
| 19 | How important it is for you that the store has employees that are consistently courteous with customers. | 1 | 2 | 3 | 4 | 5 | 6 | 7 |
| 20 | How important it is for you that the store has employees that treat customers courteously on the telephone. | 1 | 2 | 3 | 4 | 5 | 6 | 7 |
| 21 | How important it is for you that the store willingly handle returns and exchanges. | 1 | 2 | 3 | 4 | 5 | 6 | 7 |
| 22 | How important it is for you that the store shows a sincere interest in solving customer’s problem. | 1 | 2 | 3 | 4 | 5 | 6 | 7 |
| 23 | How important it is for you that the store has employees who can handle customer complaints directly and immediately. | 1 | 2 | 3 | 4 | 5 | 6 | 7 |
| 24 | How important it is for you that the store offers high quality merchandise. | 1 | 2 | 3 | 4 | 5 | 6 | 7 |
| 25 | How important it is for you that the store provides plenty of convenient parking for customers. | 1 | 2 | 3 | 4 | 5 | 6 | 7 |
| 26 | How important it is for you that the store has operating hours convenient to all their customers. | 1 | 2 | 3 | 4 | 5 | 6 | 7 |
| 27 | How important it is for you that the store accepts most major credit cards. | 1 | 2 | 3 | 4 | 5 | 6 | 7 |
| 28 | How important it is for you that the store offers its own credit card. | 1 | 2 | 3 | 4 | 5 | 6 | 7 |
