# Supplementary material for: Service quality assessment and enhancement using Kano model
Source: PLoS One. 2022 Feb 25;17(2):e0264423. doi: 10.1371/journal.pone.0264423 (PMC8880948; doi:10.1371/journal.pone.0264423)
Supplement: Attachment — Submitted filename: Response to Reviewers.docx [file pone.0264423.s003.docx]

**Response to Reviewers**

1. Please ensure that your manuscript meets PLOS ONE's style requirements, including those for file naming. The PLOS ONE style templates can be found at

<https://journals.plos.org/plosone/s/file?id=wjVg/PLOSOne_formatting_sample_main_body.pdf>  and

[https://journals.plos.org/plosone/s/file?id=ba62/PLOSOne_formatting_sample_title_authors_affiliations.pdf](about:blank)

Response:

Thank you for your comment. The manuscript is formatted and named according to the PLOS ONE’s style requirements.

2. Please include additional information regarding the survey or questionnaire used in the study and ensure that you have provided sufficient details that others could replicate the analyses. For instance, if you developed a questionnaire as part of this study and it is not under a copyright more restrictive than CC-BY, please include a copy, in both the original language and English, as Supporting Information.

Response:

Thank you for your comment. The questionnaire is included as supporting information. We also included minimal anonymized data set as supporting information necessary to replicating the analysis.

3. Please provide additional details regarding participant consent. In the ethics statement in the Methods and online submission information, please ensure that you have specified (1) whether consent was informed and (2) what type you obtained (for instance, written or verbal, and if verbal, how it was documented and witnessed). If your study included minors, state whether you obtained consent from parents or guardians. If the need for consent was waived by the ethics committee, please include this information.

If you are reporting a retrospective study of medical records or archived samples, please ensure that you have discussed whether all data were fully anonymized before you accessed them and/or whether the IRB or ethics committee waived the requirement for informed consent. If patients provided informed written consent to have data from their medical records used in research, please include this information.

Response:

Thank you for your comment. We have collected written consent in the survey while explaining the survey instruction. Screenshot of the survey instruction along with the process of collecting consent is provided below.

4. We note that you have indicated that data from this study are available upon request. PLOS only allows data to be available upon request if there are legal or ethical restrictions on sharing data publicly. For more information on unacceptable data access restrictions, please see http://journals.plos.org/plosone/s/data-availability#loc-unacceptable-data-access-restrictions.

Response:

Thank you for your comment. We included minimal anonymized data set as supporting information necessary to replicating the analysis.

In your revised cover letter, please address the following prompts:

a) If there are ethical or legal restrictions on sharing a de-identified data set, please explain them in detail (e.g., data contain potentially sensitive information, data are owned by a third-party organization, etc.) and who has imposed them (e.g., an ethics committee). Please also provide contact information for a data access committee, ethics committee, or other institutional body to which data requests may be sent.

Response:

Thank you for your comment. There are no ethical or legal restrictions on sharing a de-identified data set. We included minimal anonymized data set as supporting information necessary to replicating the analysis. For more information please provide a data request to Sharareh Kermanshachi ([sharareh.kermanshachi@uta.edu](mailto:sharareh.kermanshachi@uta.edu)).

b) If there are no restrictions, please upload the minimal anonymized data set necessary to replicate your study findings as either Supporting Information files or to a stable, public repository and provide us with the relevant URLs, DOIs, or accession numbers. For a list of acceptable repositories, please see http://journals.plos.org/plosone/s/data-availability#loc-recommended-repositories.

We will update your Data Availability statement on your behalf to reflect the information you provide.

Response:

Thank you for your comment. We included minimal anonymized data set as supporting information necessary to replicating the analysis.

Reviewers' comments:

Reviewer's Responses to Questions

**Comments to the Author**

1. Is the manuscript technically sound, and do the data support the conclusions?

The manuscript must describe a technically sound piece of scientific research with data that supports the conclusions. Experiments must have been conducted rigorously, with appropriate controls, replication, and sample sizes. The conclusions must be drawn appropriately based on the data presented.

Reviewer #1: Partly

Reviewer #2: Yes

2. Has the statistical analysis been performed appropriately and rigorously?

Reviewer #1: Yes

Reviewer #2: Yes

3. Have the authors made all data underlying the findings in their manuscript fully available?

The [PLOS Data policy](http://www.plosone.org/static/policies.action#sharing) requires authors to make all data underlying the findings described in their manuscript fully available without restriction, with rare exception (please refer to the Data Availability Statement in the manuscript PDF file). The data should be provided as part of the manuscript or its supporting information, or deposited to a public repository. For example, in addition to summary statistics, the data points behind means, medians and variance measures should be available. If there are restrictions on publicly sharing data—e.g. participant privacy or use of data from a third party—those must be specified.

Reviewer #1: Yes

Reviewer #2: Yes

4. Is the manuscript presented in an intelligible fashion and written in standard English?

PLOS ONE does not copyedit accepted manuscripts, so the language in submitted articles must be clear, correct, and unambiguous. Any typographical or grammatical errors should be corrected at revision, so please note any specific errors here.

Reviewer #1: Yes

Reviewer #2: Yes

5. Review Comments to the Author

Please use the space provided to explain your answers to the questions above. You may also include additional comments for the author, including concerns about dual publication, research ethics, or publication ethics. (Please upload your review as an attachment if it exceeds 20,000 characters)

Reviewer #1: There should be better explanation of literature gap, more details about the way that the questionnaire was conducted (time frame, and other similar details). The questionnaire should be part of the paper... There are no limitations of this research as well as suggestions for future research.

Response:

We are grateful to have this comment. This comment provides us opportunity to improve our work. Following modifications are made to address the above comments.

Page 3:

To enjoy an advantage over its competitors, a service provider must know the attributes that contribute to their customers’ satisfaction. Several studies have employed various research approaches to identifying customer satisfaction characteristics in different sectors as well as the retail sector. Kim et al. (2020) studied customer equity and customer satisfaction in traditional and new retail formats using regression analysis. In another study, Nicod et al (2020) found that providing proactive training will increase the sales value per customer but will not enhance customer satisfaction. Veloso et al (2017) established using a multi-level and hierarchical model that the customer satisfaction and service perceived quality has a significant correlation among them with respect to the retail industry. The above-mentioned studies have a limited scope in a prioritized list of characteristics to focus on when enhancing customer satisfaction in the retail store. Moreover, very little existing literature have determined such characteristics using multiple approaches simultaneously in the retail store. Hence, the aim of this study was to identify, categorize, and rank the traits of retail stores in relation to customer satisfaction. To achieve this goal, the following objectives were formulated: (1) develop a potential list of attributes that affect customer satisfaction; (2) identify the types of attributes (must-be, one-dimensional, attractive, and indifferent), based on customers’ perceptions; and (3) rank and weight the identified attributes. The findings of this study will help retail store service providers understand the relative importance of the attributes, based on the level of their impact on customer satisfaction.

Page 12:

In parallel with the functional and dysfunctional questions, a self-stated important question was included for each attribute. A scale from 1 to 7, with 1 being not at all important and 7 being extremely important, was provided to determine the relative importance of each attribute to their satisfaction level. Survey was prepared such way so that it can be completed within 15 minutes. The survey questions for this study were reviewed by a committee of experienced faculty members Eastern Mediterranean University. The questions were approved and ensured that research ethics were properly applied.

Page 22:

**Limitation**

This study demonstrated the evaluation of customer satisfaction in the retail sector, using the Kano model. It should be noted, however, that the results of this study are mainly representative of young customers, as that was the target group of the questionnaire, and they should not be generalized for customers from other age groups.

**Suggestions for future research**

The inclusion of customers from several other age groups as target participants will widen the scope of this research. Similar studies could be performed in different parts of the world with the different economic conditions of the people which will help in identifying the connection between income level and customer satisfaction characteristics for the retail sector if there is any. Similarly, this research could be replicated in sectors other than retail, for example, supply sector, to determine the characteristics to improve for better performance and satisfaction.

Survey instruction mentioning time required to complete the survey.

Reviewer #2: The goal of the article is clear.
1. An 'Abstract" should be added to recapitulate the main purpose, principal arguments, key observations and policy implications of the study.

Response:

Thank you for your comment. Following changes are made to address this comment.

Page 3:

**Abstract**

Success in the retail sector is highly dependent on customer satisfaction. Maintaining a competitive edge depends upon the service providers knowing and enacting what is important to their customers. Multiple studies have employed various research approaches to identifying characteristics of customer satisfaction in different sectors as well as retail sector. However, very few have determined such characteristics using multiple approaches simultaneously in the retail store. This study aims to identify, categorize, and rank the retail store attributes, based on their effects on customer satisfaction. A survey focusing on retail store characteristics that impact customer satisfaction was developed and distributed. Over 400 responses were collected and evaluated, using the Kano model. Results showed that visually appealing facilities and error-free transactions are of prime importance to customers. They are taken for granted, but their absence plays a significant role in customer dissatisfaction. An easy-to-navigate store layout and readily available service increase customer satisfaction, but their absence doesn’t decrease customer satisfaction. Clean public areas and modern-looking equipment are important, and improvements to them increase customer satisfaction at a proportional rate. The findings of this study will assist service providers in realizing the relative importance of the attributes of retail stores and in evaluating the impacts of their current practices on customer satisfaction levels. Such importance will help retail sector policy makers in mandating policies focusing on must-have attributes to preserve customer satisfaction.

2. There are minor linguistic and grammatical inconsistencies in the text. The manuscript would benefit from thorough editing for improvement of English.

Response:

We are grateful to have this comment. We have thoroughly read our manuscript and improved our English.
