# Supplementary material for: Service quality assessment and enhancement using Kano model
Source: PLoS One. 2022 Feb 25;17(2):e0264423. doi: 10.1371/journal.pone.0264423 (PMC8880948; doi:10.1371/journal.pone.0264423)
Supplement: Attachment — Submitted filename: Response to Reviewers.docx [file pone.0264423.s004.docx]

**Response to Reviewers**

Reviewers' comments:

Reviewer's Responses to Questions

**Comments to the Author**

1. If the authors have adequately addressed your comments raised in a previous round of review and you feel that this manuscript is now acceptable for publication, you may indicate that here to bypass the “Comments to the Author” section, enter your conflict-of-interest statement in the “Confidential to Editor” section, and submit your "Accept" recommendation.

Reviewer #1: (No Response)

2. Is the manuscript technically sound, and do the data support the conclusions?

The manuscript must describe a technically sound piece of scientific research with data that supports the conclusions. Experiments must have been conducted rigorously, with appropriate controls, replication, and sample sizes. The conclusions must be drawn appropriately based on the data presented.

Reviewer #1: Yes

3. Has the statistical analysis been performed appropriately and rigorously?

Reviewer #1: Yes

4. Have the authors made all data underlying the findings in their manuscript fully available?

The [PLOS Data policy](http://www.plosone.org/static/policies.action#sharing) requires authors to make all data underlying the findings described in their manuscript fully available without restriction, with rare exception (please refer to the Data Availability Statement in the manuscript PDF file). The data should be provided as part of the manuscript or its supporting information, or deposited to a public repository. For example, in addition to summary statistics, the data points behind means, medians and variance measures should be available. If there are restrictions on publicly sharing data—e.g. participant privacy or use of data from a third party—those must be specified.

Reviewer #1: Yes

5. Is the manuscript presented in an intelligible fashion and written in standard English?

PLOS ONE does not copyedit accepted manuscripts, so the language in submitted articles must be clear, correct, and unambiguous. Any typographical or grammatical errors should be corrected at revision, so please note any specific errors here.

Reviewer #1: Yes

6. Review Comments to the Author

Please use the space provided to explain your answers to the questions above. You may also include additional comments for the author, including concerns about dual publication, research ethics, or publication ethics. (Please upload your review as an attachment if it exceeds 20,000 characters)

Reviewer #1: Dear Authors,
Thank you for your improvements and comments. There are a couple of minor changes that should be made:
- questionnaire should be a part of the document (provided in anex part)

Response:

Thank you very much for your comment. Questionnaire is added to the appendix at the end of the manuscript document.

- when it is about the time frame, there should be described how long did the whole process of collecting data lasted (from distributing the questionnaire to the beginning of the analysis) not the time frame needed to fill the questionnaire.

Response:

Thank you for your valuable comment. The distribution of the survey took total of five months from the initial distribution of the survey till the start of the analysis. This information is added to the manuscript. The changes that are made to add this information in the manuscript is shown below.

**Page 13:**

**Pilot testing and survey distribution**

A sampling of 525 young people who were regular customers of retail stores was selected to receive the survey. Caution was given not to include any minor in this study. More than 50% were from the age group of 21-24, only 3% were older than 33, and 70% of them had a monthly income less than 4000 TL. Before conducting the survey, the questionnaire was pilot tested with 30 individuals to evaluate the survey questions prior to a larger distribution. The questionnaires were revised, based on the results of the pilot test, to reflect the purpose of the study. The pilot testing needed one month to be completed. The major distribution of the survey took four months to be completed. Hence, the distribution of the survey took total of five months from the initial distribution of the survey till the start of the analysis. The survey responses were collected by paper. Total of 400 survey responses were collected with the response rate of 76%. In the first page of the survey, respondents were notified that participation in the survey was voluntarily and their written consent was collected.

- additional information on how much questionaries were distributed, what is the response level (percentage) could be useful...

Response:

Thank you for your insightful comment. We distributed 525 surveys and collected 400 survey response. This resulted in 76% response rate. This information is also added in the manuscript. Following changes are made in the manuscript to add this information.

**Page 13:**

**Pilot testing and survey distribution**

A sampling of 525 young people who were regular customers of retail stores was selected to receive the survey. Caution was given not to include any minor in this study. More than 50% were from the age group of 21-24, only 3% were older than 33, and 70% of them had a monthly income less than 4000 TL. Before conducting the survey, the questionnaire was pilot tested with 30 individuals to evaluate the survey questions prior to a larger distribution. The questionnaires were revised, based on the results of the pilot test, to reflect the purpose of the study. The pilot testing needed one month to be completed. The major distribution of the survey took four months to be completed. Hence, the distribution of the survey took total of five months from the initial distribution of the survey till the start of the analysis. The survey responses were collected by paper. Total of 400 survey responses were collected with the response rate of 76%. In the first page of the survey, respondents were notified that participation in the survey was voluntarily and their written consent was collected.

- Limitations and Suggestions for future research should be part of the Conclusion.

Response:

Thank you for your valuable comment. The limitations and suggestions are included as part of the Conclusion instead of presenting them separately. Following changes are made in the manuscript.

**Conclusion**

To gain a competitive advantage, owners and/or managers in the retail sector need to know the attributes that are important to their customers. This study aimed not only to determine such attributes, but also to rank them so that the retailers could determine which ones were the most important. To fulfill the purpose of the study, a comprehensive literature review was conducted, 28 retail store attributes were determined, a Kano questionnaire was distributed, and Kano model was used to evaluate the attributes. It was explicated in this research that according to the respondents, the fulfillment of different attributes affects customer satisfaction at different levels. Results also indicated that a retail store should have visually appealing facilities in order to sustain satisfied customers, as this is a must-be attribute, the absence of which would significantly decrease customer satisfaction. An easy-to-navigate layout is an attribute from the attractive category that has a proportional relationship with customer satisfaction, but its absence does not cause customer dissatisfaction. Attributes such as providing clean rest areas have a proportional relationship with customer satisfaction. Based on the self-stated importance questionnaire, it was found that most of the attributes with a high level of importance are from the must-be and one-dimensional categories. The findings of this study will help service providers become more knowledgeable about the relative importance of the attributes of retail stores and will enable them to evaluate the impact of their current practices on customer satisfaction levels.

This study demonstrated the evaluation of customer satisfaction in the retail sector, using the Kano model. It should be noted, however, that the results of this study are mainly representative of young customers, as that was the target group of the questionnaire, and they should not be generalized for customers from other age groups.

The inclusion of customers from several other age groups as target participants will widen the scope of this research. Similar studies could be performed in different parts of the world with the different economic conditions of the people which will help in identifying the connection between income level and customer satisfaction characteristics for the retail sector if there is any. Similarly, this research could be replicated in sectors other than retail, for example, supply sector, to determine the characteristics to improve for better performance and satisfaction.

7. PLOS authors have the option to publish the peer review history of their article ([what does this mean?](https://journals.plos.org/plosone/s/editorial-and-peer-review-process#loc-peer-review-history)). If published, this will include your full peer review and any attached files.


If you choose “no”, your identity will remain anonymous but your review may still be made public.


**Do you want your identity to be public for this peer review?** For information about this choice, including consent withdrawal, please see our [Privacy Policy](https://www.plos.org/privacy-policy).

Reviewer #1: No
